# On the Long-Period Accuracy Behavior of Inductive and Low-Power Instrument Transformers

**DOI:** 10.3390/s20205810

**Published:** 2020-10-14

**Authors:** Alessandro Mingotti, Lorenzo Bartolomei, Lorenzo Peretto, Roberto Tinarelli

**Affiliations:** Department of Electrical, Electronic and Information Engineering, Guglielmo Marconi Alma Mater Studiorum, University of Bologna, Viale del Risorgimento 2, 40136 Bologna, Italy; lorenzo.bartolomei@unibo.it (L.B.); lorenzo.peretto@unibo.it (L.P.); roberto.tinarelli3@unibo.it (R.T.)

**Keywords:** accuracy, instrument transformers, low-power instrument transformers, voltage transformers, temperature, ratio error, phase displacement

## Abstract

The accuracy evaluation of instrument transformers is always a key task when proper control and management of the power network is required. In particular, accuracy becomes a critical aspect when the grid or the instrumentation itself is operating at conditions different from the rated ones. However, before focusing on the above non-rated conditions, it is important to fully understand the instrument transformer behavior at rated conditions. To this end, this work analyzed the accuracy behavior of legacy, inductive, and low-power voltage transformers over long periods of time. The aim was to find patterns and correlations that may be of help during the modelling or the output prediction of voltage transformers. From the results, the main differences between low-power and inductive voltage transformers were pointed out and described in detail.

## 1. Introduction

The ambitious goal of a fully decarbonized European energy supply planned for 2050 [1] requires that all involved actors work synergistically in that direction. A significant part of such a plan involves the massive diffusion and installation of renewable energy sources (RES). However, one of the drawbacks of their diffusion is the increase in grid-injected disturbances, and hence of power quality (PQ) issues [2,3,4,5], that may alter the correct operation of the grid.

A first consequence of the existence of PQ issues in the grid is the fact that proper monitoring, identification, and resolution must be implemented. Therefore, the measurement equipment, in-field deployed, to measure electrical quantities during the normal operation of the network should be also capable of working correctly during anomalous conditions.

As is well known, the devices that are meant to measure electrical quantities in the grid are the instrument transformers (ITs). They can be designed to be installed in all voltage levels (high, medium, and low voltage (HV, MV, and LV)), and they typically scale and send the measured quantities to data concentrators, acquisition systems, and cloud-based solutions.

With the introduction of RES and of a variety of intelligent electronic devices (IEDs), ITs are now required to operate in a harsher environment than in the past. In fact, they should be reliable and correctly measure electrical quantities when they are affected by all kinds of PQ issues. For example, the effects of various disturbances on ITs were assessed in [6,7,8], while authors of [9,10] evaluated how ITs contribute to the overall PQ of the network. Finally, the use of ITs for PQ evaluation was evaluated in [11].

Of course, an IT is not the only grid component that has to work in both normal and anomalous conditions. In fact, all electrical assets, such as energy meters [12,13,14], electric machines [15,16], and accessories [17,18,19], have to be properly designed to avoid (i) encountering any grid malfunctions or (ii) having corrupted and incorrect measurements sent and processed by typical algorithms dedicated to the control and management of the network [20,21,22].

In addition to what was mentioned above, it is clear that PQ, which directly affects the grid, is not the only issue related to electrical assets. In fact, external factors like temperature, humidity, electric fields, and pressure are influential quantities that modify the behavior of the ITs and, in some cases, lead to significant variation in their performance.

The literature is constantly being populated by new material trying to tackle every single aspect related to this topic. In [23,24], for example, the effect of moisture on ITs was studied. The behavior of ITs when subjected to an electric field was addressed in [25,26,27], while temperature as an influential factor was analyzed in [28,29,30].

In light of all of the above, this work aims to contribute in that direction. Its focus is on medium-voltage ITs (in particular, voltage transformers (VTs)), both the legacy and the new-generation ones, and the idea is to understand their accuracy behavior over long time intervals (compared with typical standard tests, which are single tests lasting no more than seconds/minutes). Accuracy is evaluated, according to the standards, in terms of ratio error and phase displacement. To really focus on the long time period behavior, the operating conditions of the ITs under test are the rated ones. The outcomes of this work aim to (i) provide the literature with a set of tests which was not available, (ii) help manufacturers and final users to understand the behavior of different types of VTs, and (iii) support the modelling of VTs, considering that each of them has a specific behavior over time.

The remainder of the work is structured as follows: Section 2 introduces and describes the IT scenario and the related standards. The measurement setup developed within this work is presented in Section 3, and the experimental tests and results performed and obtained by implementing this setup are contained in Section 4. Finally, Section 5 summarizes the main achievements and the conclusion of this research.

## 2. Instrument Transformers

### 2.1. Introduction and Standards

Instrument transformers are the key measurement devices for obtaining voltages and currents from the grid. In terms of technological development over the years, ITs can be distinguished into two categories: the legacy inductive ITs and the new generation of low-power ITs (LPITs), also referred to as non-conventional instrument transformers. This latter kind of transformer features smaller dimensions and weight, ease of installation in harsh environments, and larger bandwidth compared with the legacy ones. Furthermore, as the name implies, the LPIT’s output is typically one or two orders of magnitude lower (fewer volts, fewer milliamperes) than the output of an inductive IT. All these characteristics are encouraging the diffusion of LPITs in the distribution network; however, the low economic availability of distribution system operators (DSOs) to invest in meshed networks and a large portion of networks, like the medium- and low-voltage ones, makes the legacy IT a very common and reliable device that is worth being studied and deployed in the field.

All kinds of ITs are standardized by the IEC 61869 series. In particular, IEC 61869-1 [31] and -6 [32] are the general documents for legacy ITs and LPITs, respectively (both current and voltage). The remaining documents of the standard series deal with specific kinds of transformers, providing the necessary information on testing, specifications, accuracy, and definitions for final users and manufacturers. For example, IEC 61869-2 [33] and -3 [34] describe inductive current and voltage transformers (CTs and VTs), respectively, while IEC 61869-10 [35] and -11 [36] are the analogous documents for the low-power current and voltage transformers (LPCTs and LPVTs), respectively.

### 2.2. Uncertainty Evaluation

First of all, ITs must be compliant with the rated operating conditions of the power network. Two widely adopted standards that define limits and thresholds for the grid quantities are the EN 50160 [37] and the IEEE 519 [38], in which rated and distorted conditions of the grid are considered. Second, each IT is associated with an accuracy class (AC) that defines its performance.

As is well known, accuracy is evaluated by means of two parameters, the ratio error *ε* and the phase displacement Δ*φ*, which are defined as follows:

(1) $$\varepsilon =\frac{k_{r}V_{2}-V_{1}}{V_{1}}\times 100\%$$

(2) $$\Delta \varphi ={\hat{V}}_{2}-{\hat{V}}_{1}$$

where $V_{1}$ and $V_{2}$ are the rms values of the 50 Hz component of the primary and secondary voltages, respectively, and $k_{r}$ is the rated transformation ratio of the device under test. Finally, ${\hat{V}}_{1}$ and ${\hat{V}}_{2}$ are the 50 Hz phase components of the primary and secondary voltages, respectively.

In what follows, *ε* and Δ*φ* are the two parameters monitored to understand the accuracy behavior of the ITs in a long time interval. In addition, each *ε* and Δ*φ* computation is associated with a temperature measurement of the working environment.

### 2.3. Types of VTs

In Section 2.1, a first distinction among ITs was provided. In this section, more details are given about the VTs used and tested in this work.

**Figure 1:**
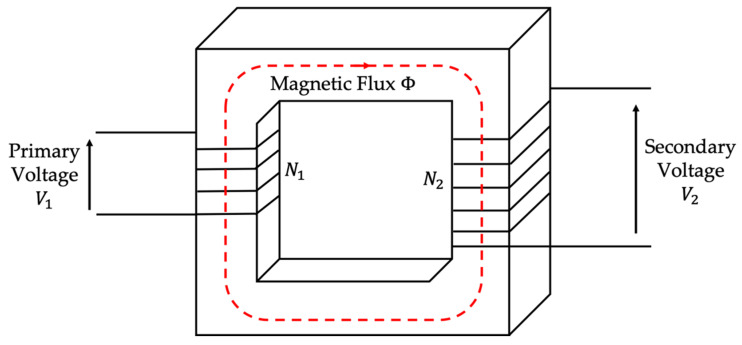
Basic schematic of an inductive transformer.

The most common VT is the well-known inductive one, which is schematized in Figure 1. It consists of a metallic, magnetic core and of two copper windings referred to as primary and secondary. Each winding has a specific number of turns ($N_{1}$ and $N_{2}$ in the picture) that makes it possible to obtain the desired transformer ratio. The inductive VT working principle is based on two famous laws: Faraday’s and Lenz’s. This way, a scaled secondary voltage can be obtained starting from a fixed primary voltage. In particular, it is

(3) $$\frac{N_{1}}{N_{2}}=\frac{V_{1}}{V_{2}}$$

As for its operation, the inductive VT is affected by some loss sources, which can be due to the Joule effect, leakage flux, eddy currents, and hysteresis (not detailed here because this is out of the scope of the work).

**Figure 2:**
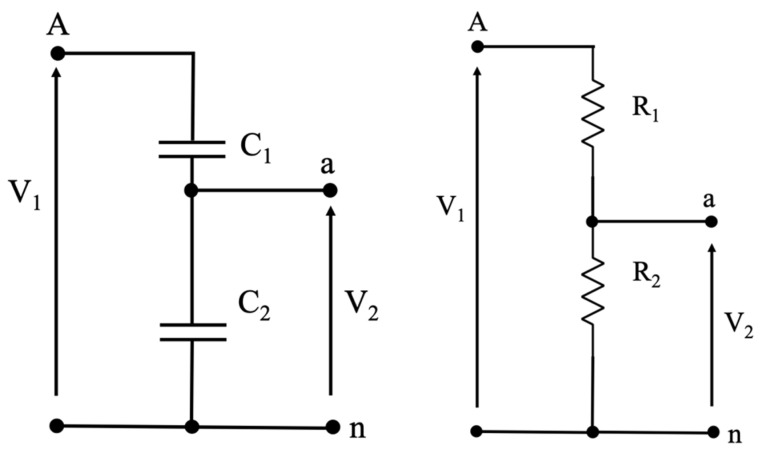
Working principle of a capacitive (**left**) and a resistive (**right**) low-power voltage transformer (LPVT).

Turning to the new generation of VTs, the LPVTs, they are often designed with a working principle that is not correlated with the induction laws. In fact, two of the most common types of LPVTs are the resistive and the capacitive ones. Both their schematics are depicted in Figure 2 and, as it can be noted, both LPVTs rely on the voltage divider principle.

The simplest one is the resistive LPVT. It consists of two resistors on which $V_{1}$ is divided. Therefore, $R_{1}$ is a very high resistor compared to $R_{2}$, and the ratio is fixed according to the requirements of the device connected to the secondary terminals of the LPVT. In terms of accuracy, $R_{1}$ is typically less accurate due to its high-voltage nature, and hence it is more complicated and expensive to build. The input–output relation of a resistive LPVT is as follows:

(4) $$V_{2}=V_{1}\frac{R_{2}}{R_{1}+R_{2}}$$

As for capacitive LPVTs, once again the voltage divider principle applies, but it takes into account the behavior of a capacitor, which is substantially opposite to the resistor. In fact, looking at Figure 2, it can be seen that $C_{2}$ is the biggest capacitor, subjected to the lowest voltage part of $V_{1}$. In terms of accuracy, $C_{2}$ is usually a commercial capacitor, while $C_{1}$ can be obtained from the body of the LPVT, hence from the resin or other insulating materials that comprise its case. Consequently, there is not much control over the insulating material accuracy; hence it is the less accurate capacitor of the two. The input–output relation of a capacitive LPVT is as follows:

(5) $$V_{2}=V_{1}\frac{C_{1}}{C_{1}+C_{2}}$$

## 3. Experimental Measurement Setup

**Figure 3:**
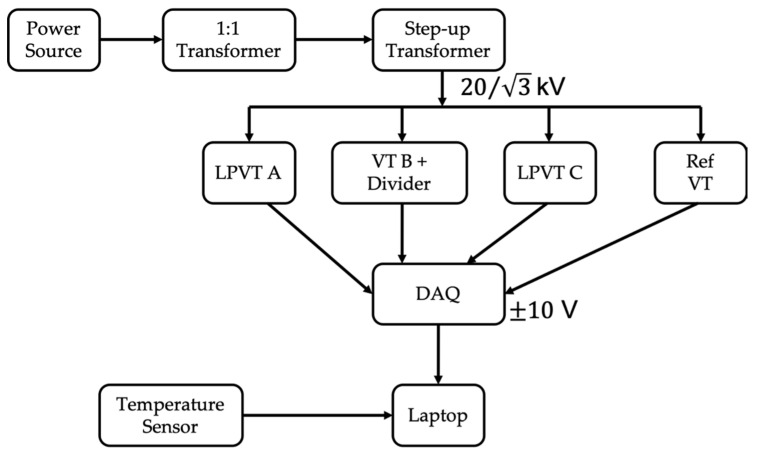
Schematic representation of the measurement setup.

The measurement setup developed to perform the experimental tests is presented in Figure 3.

It consists of the following:

- An Agilent 6813B power source to feed the insulating and the step-up transformers. It features a max rms voltage of 300 V and a max power of 1750 VA.
- An insulating transformer with 1:1 ratio. Its main purpose is to provide galvanic insulation between the low- and the medium-voltage sides.
- A step-up transformer with 1:142 ratio. It guarantees a stable 20/√3 kV voltage at the terminals of the transformers under test.
- A reference capacitive-resistive VT used to measure the rated voltage that has to be used in the accuracy computations. It features a 5981:1 ratio and an accuracy of 0.03% on the ratio and 0.3 mrad on the phase, according to its calibration certificate.
- Three off-the-shelf VTs under test. Two are LPVTs, while the third is a classical inductive VT. Their main characteristics are listed in Table 1. In particular, the type, the primary and the secondary rated voltages ($V_{1R}$ and $V_{2R}$), and the accuracy class (AC) of the VTs are reported.
- A pure-resistive voltage divider, previously characterized, has been used to reduce the secondary voltage of B to a level suitable for the acquisition system. The divider ratio is 100:1 and it is composed of high-precision resistors that can be considered insensitive to temperature variations (few ppm/°C).
- An NI9239 data acquisition board (DAQ) to collect the secondary voltages of the three transformers under test plus the secondary voltage of the reference one. The DAQ features a full scale of ±10 V, a 24-bit architecture, 50 kSa/s per channel of maximum acquisition rate, and gain and offset errors of ±0.03% and ±0.008%, respectively.
- An NCT75 programmable temperature sensor, the characteristics of which are listed in Table 2. The sensor was used to measure the ambient temperature at which the transformers were operating.

To summarize, the experimental setup was developed to feed the transformers under test with their rated voltage (20/√3 kV) and then to measure and acquire their secondary voltages plus the reference one. In addition, all the measurements are associated with the ambient temperature at which they were collected.

## 4. Experimental Tests and Results

### 4.1. Experimental Tests

The main idea of the work was to understand the accuracy behavior of the LPVTs and classical VTs over time when they operate at rated conditions of voltage, frequency, and temperature. The behavior was evaluated in what was called a “long time” interval because, compared to the typical seconds/minutes-long tests described by the standards, what was performed in this work can be considered long. However, even if long tests were performed, they were not considered aging tests because that was not the purpose. Therefore, using the measurement setup described in Section 3, the test consists of feeding the three VTs at 20/√3 kV, 50 Hz, and uncontrolled temperature (but within typical laboratory values, 25 to 27 °C) for 12 days. Within such a time range, every 4 h, 100 measurements of ratio error and phase displacement were performed together with the temperature measurement of the laboratory. Afterwards, the mean value of the 100 measurements and the standard deviation of *ε* and Δ*φ* were computed and collected to be further processed and assessed.

### 4.2. Experimental Results

As previously anticipated, the experimental results are given in terms of *ε* and Δ*φ* for the three VTs under test. Furthermore, such results are associated with their standard deviation of the mean.

**Figure 4:**
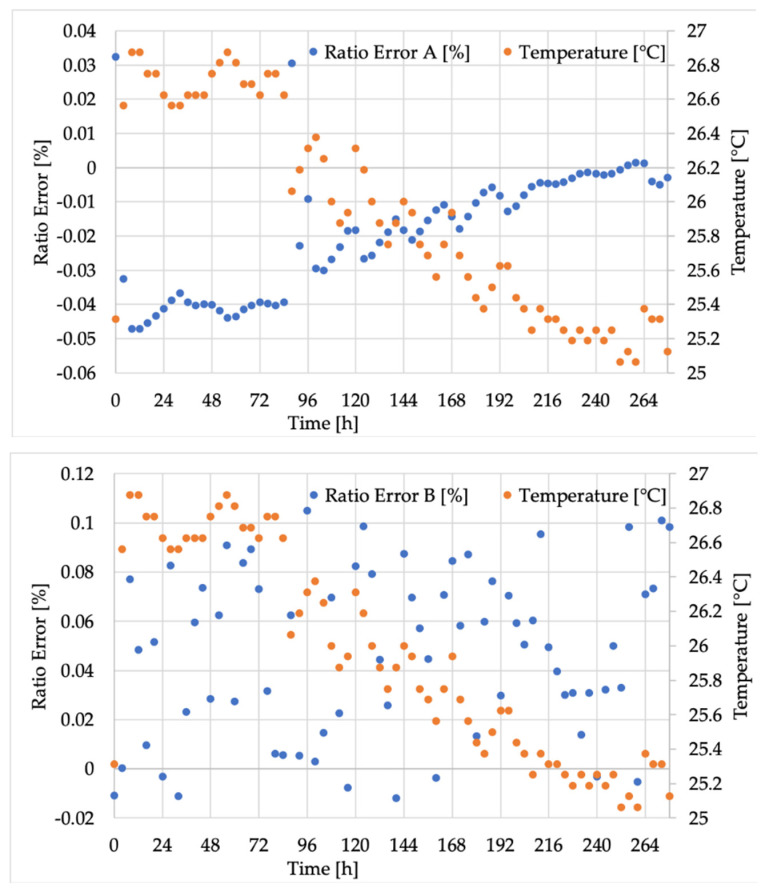

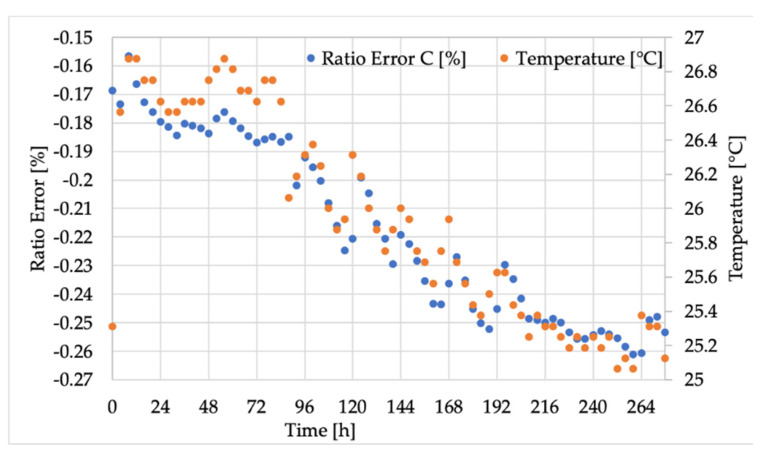
Ratio error results for VTs (**A**–**C**) (from top to bottom).

Starting from *ε*, Figure 4 shows the results obtained from measurements on VTs A, B, and C. The graphs contain *ε* (in %) and the temperature (in °C) on the left and right y-axes, respectively. The two quantities are also differentiated by using blue for *ε* and orange for temperature. On the x-axes, the time expressed in hours can be found. Considering that the adopted unit for the x-axis is 24 h, in every section of the graphs, one day of measurements can be seen.

**Figure 5:**
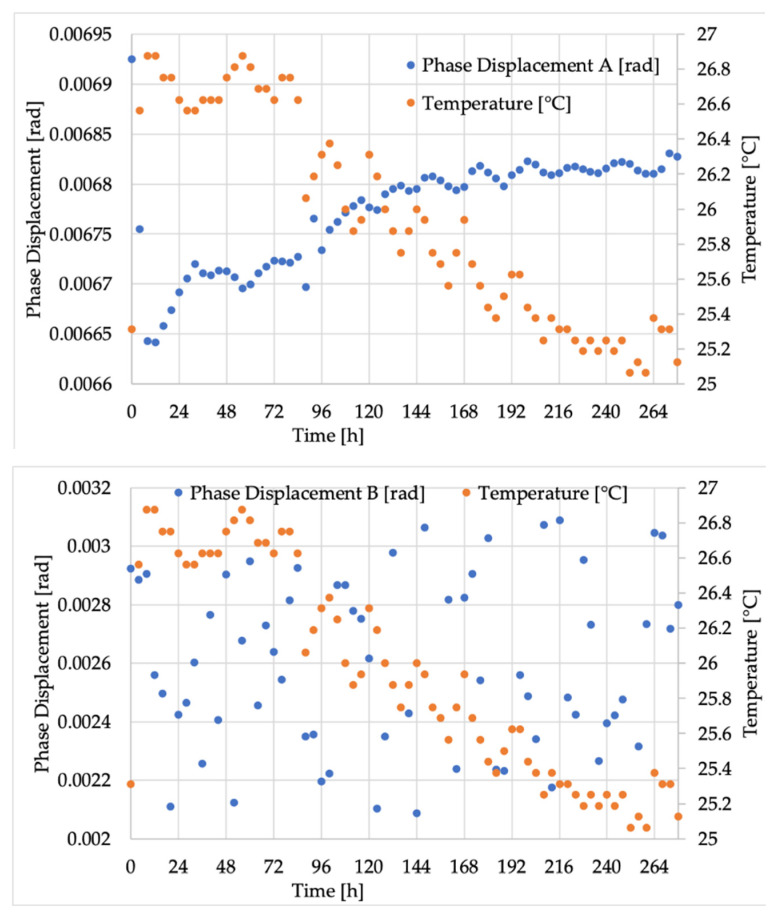

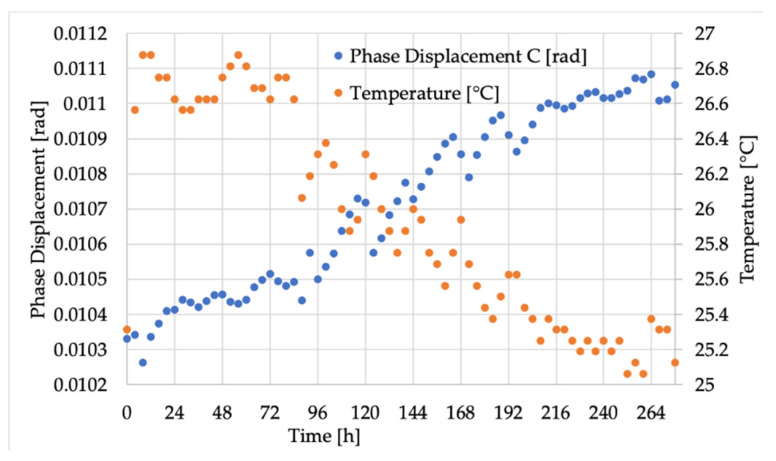
Phase displacement for VTs (**A**–**C**) (from top to bottom).

Analogously, the results for Δ*φ* (measured in rad) are provided in Figure 5.

### 4.3. Discussion

The first comment that has to be made is that the three VTs were working properly within their accuracy class limits (Table 1). To better see that, Table 3 contains the measured minimum, maximum, and overall variation of *ε* and Δ*φ* (*dε* and Δ*φ*, respectively) over the entire duration of the test.

The values in the table are reported with a number of significant digits that takes into account the standard deviation of the mean associated with the measurements from each VT. From a cursory comparison of the three VTs, it can be concluded that VT A (the resistive one) showed less disperse ε and Δφ values, whereas B (the inductive VT), had the worst performance. In detail, the maximum mean standard deviation observed for ε and Δφ were in the order of 10^−7^ and 10^−9^, 10^−4^ and 10^−7^, and 10^−6^ and 10^−8^ for A, B, and C, respectively. However, the accuracy level obtained for all the VTs was appropriate for the type of test and study performed on them. To complete the discussion about the uncertainty related to results, it is worth highlighting that the uncertainty introduced by the measurement setup was omitted to avoid any confusion (however, it was in the order of 10^−5^ and 10^−4^ for *ε* and Δ*φ*, respectively, for B). The reason for this is that measurements were performed with the same setup; hence any contribution that it introduced affected all measurements in the same way.

The second comment concerns the general trend of the graphs. In fact, what clearly emerged from both Figure 4 and Figure 5, hence for both ε and Δφ, is that the inductive VT values were completely stochastic, while the low-power ones (A and C) followed the temperature profile with different laws. To validate the previous statement, the correlation coefficients of the three VTs, which linked both ε and Δφ with the temperature (T), were computed and collected in Table 4. As it emerges from the table, temperature was totally uncorrelated with ε and Δφ in the case of the inductive B, while an almost complete correlation was found for A and C and for both ε and Δ*φ*. In addition, the obtained coefficients can be considered statistically significantly different from the other parameters computed and listed in Table 4. In fact, it contains the *p*-value and the 95% confidence interval (CI) limits for each of the provided coefficients. 

**Figure 6:**
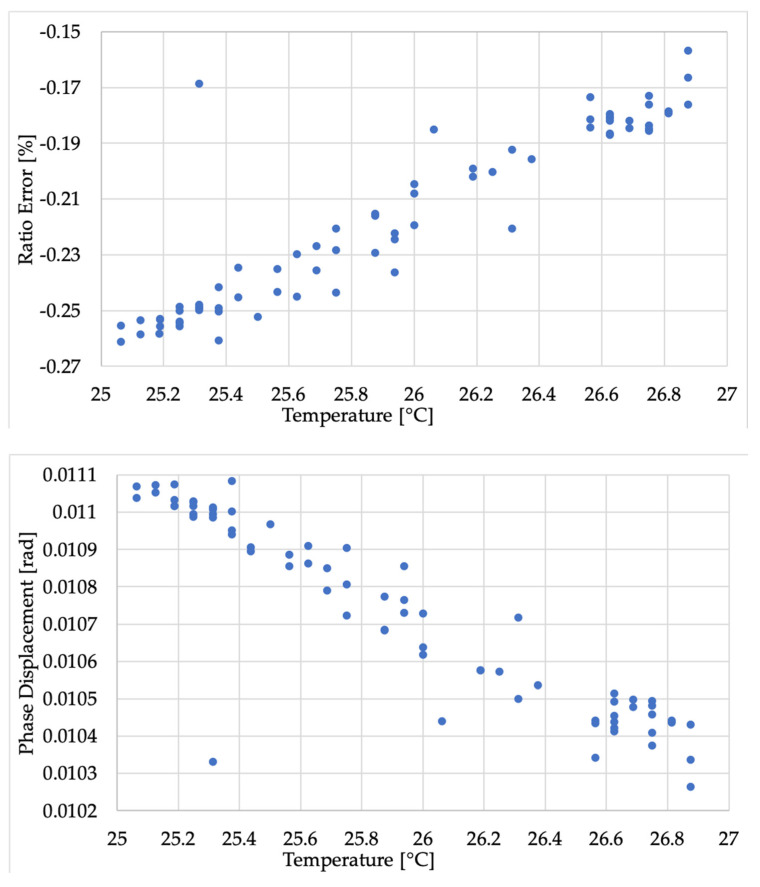
Correlation graphs between *ε* and T (**top**) and Δ*φ* and *T* (**bottom**) for VT C.

As an example, Figure 6 shows the correlation between ε and Δφ and the temperature for VT C.

Focusing now on the patterns of transformers A and C, which follow the temperature profile, the causes have to be explained. For both VTs, the explanation lies in their design and building methodology. In particular, transformer A, the resistive one, consists of a high-voltage resistor (see $R_{1}$ in Figure 2) that often features worse performance than the low-voltage resistor due to the high costs associated with the high-voltage technology. Therefore, resistor $R_{1}$ is more affected by temperature, causing a variation of the transformer ratio. To be more specific, as it can be seen in the upper graph of Figure 4, ε is negative and keeps decreasing when the temperature increases. In fact, if, as expected, $R_{1}$ increases more than $R_{2}$ with the temperature, the dominant ratio $R_{1}/R_{2}$ of Equation (4) increases. Consequently, the actual transformation ratio increases compared to $k_{r}$, resulting in a further decrease in the computed quantity ε.

Similar reasoning can be used for transformer C, the capacitive one. This time, referring to the left scheme of Figure 2, the biggest and more accurate capacitor is $C_{2}$, and the voltage is measured on its terminals. As for $C_{1}$, what is typically done is that it is obtained from the case-shell of the capacitor, as mentioned before; hence the technical limitations and the need to withstand high voltages result in a less accurate $C_{1}$. Linking this behavior with what was obtained in the bottom graph of Figure 4, it can be noted that a negative ε increases with an increase in temperature. In other words, the temperature affects the dielectric material properties of $C_{1}$ increasing its value, hence reducing the ratio $C_{2}/C_{1}$ and then leading to the measured effect.

Turning to the phase displacement, from all graphs and from Table 3, it is clear that very small variations were measured (fraction of mrad). For VT B, the behavior was the same as ε, hence displaying a stochastic spread of the measured values over time. Furthermore, even for VTs A and C, that which was observed for ε could also be extended for Δ*φ*. In fact, Δ*φ* is inversely proportional to the temperature.

From the above discussion, some relevant conclusions are as follows:

- In the considered short temperature range, it is possible to conclude that the accuracy of inductive VTs was not affected by temperature. However, it is not automatically true that this conclusion holds for wider ranges of temperature.
- Over long time intervals, the developed setup allowed even the tiniest variations of *ε* and Δ*φ* due to very small changes in the ambient temperature to be seen. As a result, such slight temperature variations had a visible effect on the LPVT’s accuracy (even if absolutely moderate). The same could not be stated for the inductive ones.
- The different ways of spreading *ε* and Δ*φ* values between LPVTs and legacy ITs raises a significant issue: when modelling them, it is not possible to simulate their behavior in the same way, even at rated conditions.
- What was observed for LPVT might be a strength or a drawback of this new generation of transformers. However, what is clear is that, considering their diffusion among distribution networks, such information has to be considered when choosing the technology to be installed in a particular operating environment.
- What was observed at rated conditions and at an ambient temperature that varies not more than a couple of degrees reinforced the studies on the influential quantities affecting the behavior, and hence the accuracy, of all kind of ITs.
- The different behaviors recorded for classical VTs and LPVTs highlight the need to differentiate the modelling of such transformers. Therefore, general models should be particularized for the specific VT that is going to be used in the considered application.

## 5. Conclusions

This paper aimed to tackle the accuracy behavior of voltage transformers during long time intervals. To this end, after introducing various voltage transformers and their properties, a measurement setup was introduced. This setup was used to assess the accuracy of three off-the-shelf voltage transformers (capacitive, inductive, and resistive) during a 12-day period. The test was performed at rated conditions but without full temperature control. The results assessment was performed in terms of two widely adopted indices, the ratio error and the phase displacement. The results revealed a huge difference between the behavior of the legacy, inductive, and low-power transformers. These results highlighted how the design of the low-power type influenced its performance, even at rated conditions, while the same did not apply for the inductive type. A strictly related consequence of that is the need to tackle the modelling and the behavior prediction of voltage transformers depending on their design and working principle.

## Figures and Tables

**Table 1:** Main characteristics of the three voltage transformers (VTs) under test.

LPVT	Type	$V_{1R}\;(V)$	$V_{2R}\;(V)$	AC
A	LPVT—Resistive	$20,000/\sqrt{3}$	$3.25/\sqrt{3}$	0.5
B	VT—Inductive	$20,000/\sqrt{3}$	133$/\sqrt{3}$	0.5
C	LPVT—Capacitive	$20,000/\sqrt{3}$	2.27$/\sqrt{3}$	0.5

**Table 2:** Main characteristics of the NCT75 temperature sensor.

Feature–Value			
Resolution	12 bits	Accuracy	±1 °C
Input Voltage	3 V to 5.5 V	Update Rate	80 ms
Temperature Range	−55 to 125 °C		

**Table 3:** Minimum, maximum, and variation of *ε* and Δ*φ* during the 12-day test.

	*ε* [%]			Δ*φ* [rad]		
	min	max	d*ε*	min	max	dΔ*φ*
A	−0.0471369	0.0323216	0.0794585	0.006641747	0.006925065	0.000283318
B	−0.0119	0.1051	0.1170	0.0018892	0.0030884	0.0011992
C	−0.261155	−0.156676	0.104479	0.01026314	0.01108411	0.00082096

**Table 4:** Correlation coefficients and statistical parameters for all VTs and both parameters *ε* and Δ*φ*.

	*ε*–*T* [-]	*p*-Value	95% CI min	95% CI max	Δ*φ–T* [-]	*p*-Value	95% CI min	95% CI max
A	−0.873	<0.0001	−0.92	−0.803	−0.898	<0.0001	−0.936	−0.841
B	−0.011	0.930	−0.245	−0.225	0.021	0.864	−0.215	−0.255
C	0.922	<0.0001	0.877	0.951	−0.919	<0.0001	−0.949	−0.873
